# Advanced Glycation End Products Impair Glucose-Stimulated Insulin Secretion of a Pancreatic *β*-Cell Line INS-1-3 by Disturbance of Microtubule Cytoskeleton via p38/MAPK Activation

**DOI:** 10.1155/2016/9073037

**Published:** 2016-08-22

**Authors:** Jia You, Zai Wang, Shiqing Xu, Wenjian Zhang, Qing Fang, Honglin Liu, Liang Peng, Tingting Deng, Jinning Lou

**Affiliations:** Institute of Clinical Medical Sciences, China-Japan Friendship Hospital, Beijing 100029, China

## Abstract

Advanced glycation end products (AGEs) are believed to be involved in diverse complications of diabetes mellitus. Overexposure to AGEs of pancreatic *β*-cells leads to decreased insulin secretion and cell apoptosis. Here, to understand the cytotoxicity of AGEs to pancreatic *β*-cells, we used INS-1-3 cells as a *β*-cell model to address this question, which was a subclone of INS-1 cells and exhibited high level of insulin expression and high sensitivity to glucose stimulation. Exposed to large dose of AGEs, even though more insulin was synthesized, its secretion was significantly reduced from INS-1-3 cells. Further, AGEs treatment led to a time-dependent increase of depolymerized microtubules, which was accompanied by an increase of activated p38/MAPK in INS-1-3 cells. Pharmacological inhibition of p38/MAPK by SB202190 reversed microtubule depolymerization to a stabilized polymerization status but could not rescue the reduction of insulin release caused by AGEs. Taken together, these results suggest a novel role of AGEs-induced impairment of insulin secretion, which is partially due to a disturbance of microtubule dynamics that resulted from an activation of the p38/MAPK pathway.

## 1. Introduction

Advanced glycation end products (AGEs) are a group of irreversible adducts from nonenzymatic reaction of reducing sugars with the free amino groups of proteins as well as lipids and nucleic acids. These harmful substances may affect the development of many degenerative diseases, including atherosclerosis, chronic renal failure, Alzheimer's disease, and diabetes mellitus [1]. In diabetic conditions, prolonged hyperglycemia leads to a significantly increased serum level of AGEs. AGEs have been implicated to play a causative role in the development of diabetes and diabetic complications [2, 3]. Recent studies showed that AGEs lower the insulin synthesis and secretion in the pancreatic *β*-cells and induce cell apoptosis [4–9]. However, the cellular mechanism of AGEs-induced *β*-cell damage is poorly understood.

Dysfunction of glucose-stimulated insulin secretion (GSIS) is one of the most important factors in the etiology of type 2 diabetes mellitus (T2DM). Insulin secretion upon glucose stimulation is biphasic, including the transient initiation phase releasing the rapid release pool of insulin granules stored close to the cellular membrane and sustained second phase that transports the reserve pool of insulin vesicles to the cellular membrane for exocytosis. The microtubule network serves as essential cytoskeleton scaffold for the transportation of insulin-containing vesicles [10, 11]. Driven by a microtubule motor protein kinesin-1, the insulin-containing vesicles move along the microtubules towards the cellular membranes [12]. The drugs interfering with microtubule polymerization reduce vesicle movement and insulin secretion [13, 14]. The microtubule stabilizing agent taxol also impairs GSIS in rodent islets [10]. Interestingly, glycemic fluctuation affects the dynamics of microtubule network in *β*-cells [15]. These results suggested that the dynamic polymerization of microtubule cytoskeleton was required for GSIS process.

The arrangement of microtubules is regulated by several cellular signaling pathways. Mitogen-activated protein kinases (MAPK) are protein kinases that contribute to direct cellular responses to various stimuli. p38/MAPK, a member of the MAPK superfamily that transduces extracellular responses, is known for its regulatory roles in apoptosis, cytokine production, transcriptional regulation, and cytoskeletal reorganization [16, 17]. p38/MAPK can be phosphorylated by high concentration of glucose or AGEs [8, 18, 19]. Additionally, the activated p38/MAPK disrupts the microtubules by changing the phosphorylation levels of the microtubule-associated proteins in hypoxic cardiomyocytes and HeLa cells [20]. Meanwhile, pharmacological inhibition of p38/MAPK facilitates microtubule polymerization [20, 21]. However, it is not known that whether AGEs-activated p38/MAPK affects the microtubule cytoskeleton in pancreatic *β*-cells, which would compromise the insulin secretion.

INS-1 cells were derived from X-ray induced rat insulinoma cells, which were employed as a *β*-cell model for their capacity of GSIS [22]. However, their heterogeneous natures and unexpected glucagon expression make it more restrictive to be used as a substitution of primary *β*-cells [23]. We previously obtained a subclone of INS-1, named as INS-1-3, by single cell cloning. Compared to INS-1 cells, INS-1-3 expressed higher level of insulin but minimum amount of glucagon and had commendable insulin secretion capacity in response to glucose stimulation and thus was considered to be a more appropriate *β*-cell model [24]. In the current study, we used INS-1-3 to investigate the mechanisms of AGEs-impaired insulin secretion. We first confirmed that AGEs caused a reduction of glucose-induced insulin secretion on our cell model. Then we addressed three questions in this study: (1) whether AGEs disturb the dynamics of microtubule network; (2) whether AGEs stimulate p38/MAPK activation; and (3) whether activated p38/MAPK leads to disturbance of microtubule dynamics.

## 2. Materials and Methods

### 2.1. Cell Culture

INS-1 cells (a gift from Dr. C. Wollheim, Geneva, Switzerland) were subcloned by limited dilution method as previously described [24]. Briefly, single colonies were identified two weeks later under microscope. INS-1-3 was picked from total of 46 single colonies for its robust expression of insulin and minimum glucagon expression. INS-1-3 cells were cultured in RPMI 1640 (GIBICO, USA) medium supplemented with 10% (v/v) fetal calf serum (Sigma-Aldrich, USA), 50 *μ*M *β*-mercaptoethanol, 10 mM HEPES, 2 mM glutamine, 1 mM sodium pyruvate, 100 units/mL penicillin, 100 *μ*g/mL streptomycin at 37°C, and 5% CO_2_. Different concentrations of BSA-AGEs (Abcam, UK) were added to the culture medium of INS-1-3 cells for various incubation time periods as indicated. BSA (Sigma) at the same concentrations as BSA-AGEs was used as controls. To study the involvement of p38/MAPK pathway in AGE-induced microtubule depolymerization, the cells were preincubated with a p38/MAPK inhibitor SB202190 (Selleckchem) at 50 *μ*M for one hour before BSA-AGEs were added in the culture medium.

### 2.2. Real-Time PCR

Total RNA was isolated using an RNA isolation kit (Qiagen, USA), which was then transcribed into cDNA using a total RNA transcription system. Real-time quantitative PCR was performed using the SYBR Green PCR Master Mix on ABI Prism 7500 Sequence Detection System (Applied Biosystems, USA). All data were analyzed using the expression of *β*-actin as a reference. Data were analyzed using the 2^−ΔΔCt^ method. The following primers were used: insulin: forward: TGCCCAGGCTTTTGTCAAACAGCACCTT and reverse: CTCCAGTGCCAAGGTCTGAA; glucagon: forward: ATAGCTGAGGAACTTGGGCG and reverse: CCAAGTGACTGGCAGGAGAT.

### 2.3. Insulin Content Measurement

INS-1-3 cells were seeded in 24-well plates with different concentration of AGEs in the culture media as indicated. After 48 hours, cells were washed twice with PBS (pH 7.4) and extracted with acid/ethanol (0.15 M HCl in 75% ethanol) overnight at 4°C. Then supernatants containing total amount of synthesized insulin were collected. The insulin concentration was measured by a rat insulin ELISA kit (ALPCO, USA). The results were normalized to the total protein concentration measured by a BCA protein assay kit (Beyotime, China).

### 2.4. GSIS Assay

INS-1-3 cells were seeded in 24-well plates and incubated for 48 hours in the experimental media containing 5.5 mM glucose and different concentrations of AGEs as indicated. After preincubation for one hour in HEPES-balanced Krebs-Ringer bicarbonate buffer (KRBH) containing 2.5 mM glucose at 37°C, the cells were challenged with 2.5 or 25 mM glucose for 40 minutes. Then the supernatants were collected for subsequent determination of insulin concentrations by a rat insulin ELISA kit (ALPCO, USA).

### 2.5. Immunoblot Analysis

INS-1-3 cells cultured in different conditions were lysed with ice-cold radioimmunoprecipitation assay (RIPA) buffer containing protease inhibitors (Roche, USA) and phosphatase inhibitors (Cell Signaling Technology, USA). Total protein concentrations were determined by a BCA protein assay kit (Beyotime, China). Aliquots of 30 *μ*g proteins were loaded on 10% SDS-polyacrylamide electrophoresis and then transferred onto PVDF membranes. Primary antibodies used were anti-p38 (1 : 1000; CST, USA), anti-phospho-p38 (1 : 1000; CST, USA), and anti-*β*-actin (1 : 20000; Sigma, USA). *β*-actin was used for normalization of protein loading. Free and polymerized tubulin fractions were probed with anti-*α*-tubulin (1 : 200; Santa Cruz, USA). Band intensities were quantified by densitometry using the NIH program Image J®.

### 2.6. Immunofluorescence

Cells were seeded on laminin-coated glass coverslips and cultured as described above. Cells were then fixed in 4% paraformaldehyde for 20 minutes, permeabilized with 0.1% Triton X-100/PBS for 8 minutes, and then blocked for 20 minutes with 1% BSA/PBS at room temperature. The cells were incubated with anti-*α*-tubulin antibody (1 : 1500; Sigma, USA) overnight at 4°C. After washing with PBS containing 1% Tween-20, the cells were stained with Alex488-labeled secondary antibody (Invitrogen, USA) for 30 minutes at 37°C. The nuclei were stained with 4′,-6-diamidino-2-phenylindole (DAPI) (Sigma, USA) for 5 minutes. Cells were imaged with confocal microscopy (TCS-P8; Leica, Germany).

### 2.7. Tubulin Polymerization Assay

Free and polymerized tubulin from cell lysates were separated by centrifugation as described previously [25, 26]. Briefly, INS-1-3 cells were washed twice with PBS before being lysed with hypotonic buffer (1 mM MgCl_2_, 2 mM EGTA, 0.5% NP40, 2 mM phenylmethylsulfonyl fluoride, 10 *μ*L protease inhibitor mixture (Sigma, USA), and 20 mM Tris-HCl, pH 6.8) for 5 minutes at 37°C in the dark. Then the cell lysates were centrifuged at 14,000 rpm for 10 minutes at room temperature. The supernatant containing the soluble tubulin was transferred to a new tube. The pellet containing the polymerized tubulin was then resuspended in the hypotonic buffer. Soluble and polymerized tubulin amounts were quantified by immunoblots. Results were calculated as a percentage of the polymerized tubulin amount to the total tubulin amount (sum of soluble and polymerized tubulin amounts).

### 2.8. Statistical Analysis

Results were expressed as the mean ± SD. The analysis was conducted with SPSS11.0 software, using unpaired Student's* t*-test for comparison between two groups and variance (ANOVA) for comparisons among multiple groups. It was considered statistically significant if *p* < 0.05.

## 3. Results

### 3.1. The Insulin Expression and Secretion in INS-1-3

**Figure 1:**
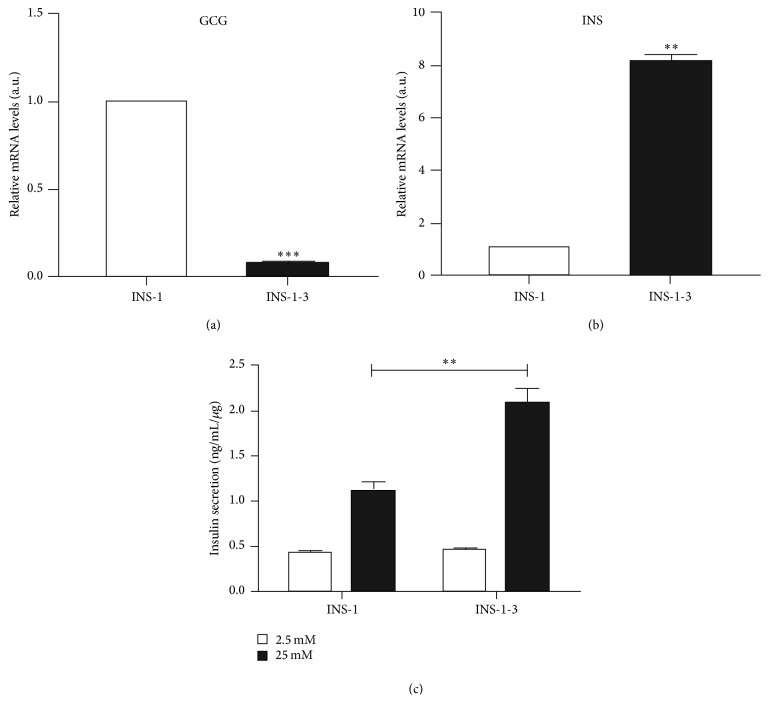
The insulin expression and secretion in INS-1-3. (a) The mRNA level of glucagon was compared between INS-1-3 and INS-1 cells, by real-time PCR. (b) The mRNA level of insulin was compared between INS-1-3 and INS-1 cells. The *β*-actin mRNA level was used as control. (c) Insulin secretion from INS-1-3 and INS-1 cells in response to 2.5 mM and 25 mM glucose, normalized to the total insulin content, measured by ELISA. Data are derived from three independent experiments and shown as mean ± SD. ^*∗∗*^ *p* < 0.01; ^*∗∗∗*^ *p* < 0.001 versus INS-1.

INS-1 cell line was isolated from an X-ray-induced rat transplantable insulinoma which is a mixture of endocrine cells with different phenotypic features [22]. We isolated and selected INS-1-3, which showed 8-fold higher insulin expression level than INS-1, with little glucagon expression detected (Figures 1(a) and 1(b)). In addition, the glucose-induced insulin secretion was two-fold higher than that in INS-1 cells (Figure 1(c)). Therefore, INS-1-3 was considered to be a better model to study the function and pathophysiology of pancreatic *β*-cells.

### 3.2. Effects of AGEs on Insulin Expression and Secretion in INS-1-3

**Figure 2:**
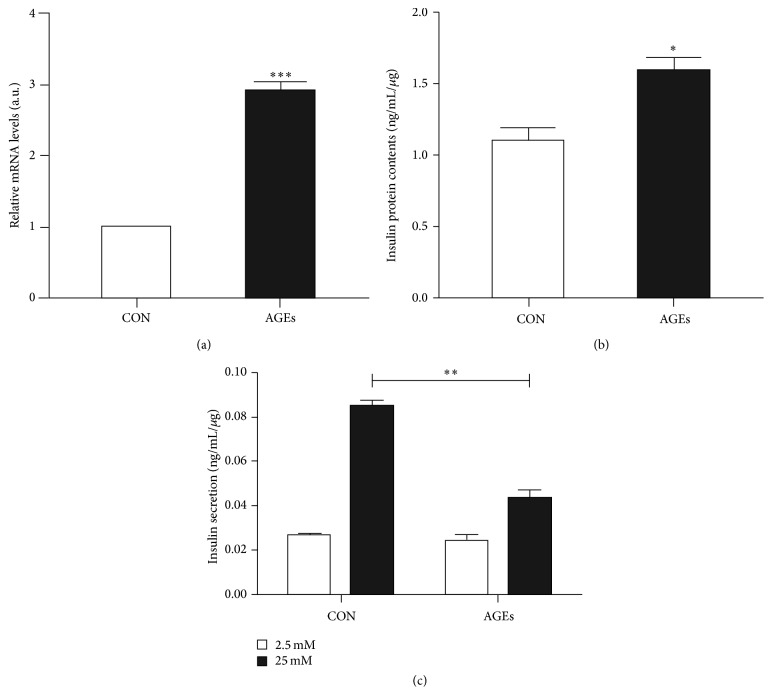
The effect of AGEs on insulin secretion in INS-1-3. (a) The mRNA level of insulin in INS-1-3 cells in the presence or absence of AGEs (200 *μ*g/mL, 48 hours), measured by real-time PCR. (b) The total intracellular insulin content in INS-1-3 in the presence or absence of AGEs (200 *μ*g/mL, 48 hours). Insulin was extracted with acid ethanol and quantified by ELISA. (c) Secreted insulin amounts in INS-1-3 cells in response to 2.5 mM and 25 mM glucose. INS-1-3 cells were incubated with or without AGEs (200 *μ*g/mL) for 48 hours. Secreted insulin levels were normalized to the total insulin content. Data are derived from three independent experiments and shown as mean ± SD. ^*∗*^ *p* < 0.05; ^*∗∗*^ *p* < 0.01; ^*∗∗∗*^ *p* < 0.001 versus control.

To determine whether AGEs affect insulin expression and secretion, INS-1-3 cells were cultured in medium with or without 200 *μ*g/mL AGE for 48 hours. Expression of insulin was measured by its mRNA and protein levels. As shown by real-time PCR, insulin mRNA level increased 2.9-fold upon AGEs treatment (Figure 2(a)). Additionally, total intracellular insulin protein amount was about 1.5-fold higher comparing AGEs-treated cells to untreated cells (Figure 2(b)). These results indicated that AGEs did not impair but enhanced the insulin synthesis in INS-1-3 cells.

To test whether the glucose response of INS-1-3 cells was affected by AGEs, we measured the GSIS with or without AGEs exposure. Without exposure to AGEs, the increase of glucose concentration from 2.5 mM to 25 mM led to a 3.3-fold increase of secreted insulin from INS-1-3 cells. However, with AGEs exposure, the cells only showed 1.8-fold increase of secreted insulin, while the basal secretion level of insulin in 2.5 mM glucose condition was not significantly affected (Figure 2(c)). This result showed that exposure to AGEs dampened the *β*-cells function of INS-1-3 cells for glucose-stimulated insulin secretion.

### 3.3. AGEs Depolymerize the Microtubule Network in INS-1-3 Cells

**Figure 3:**
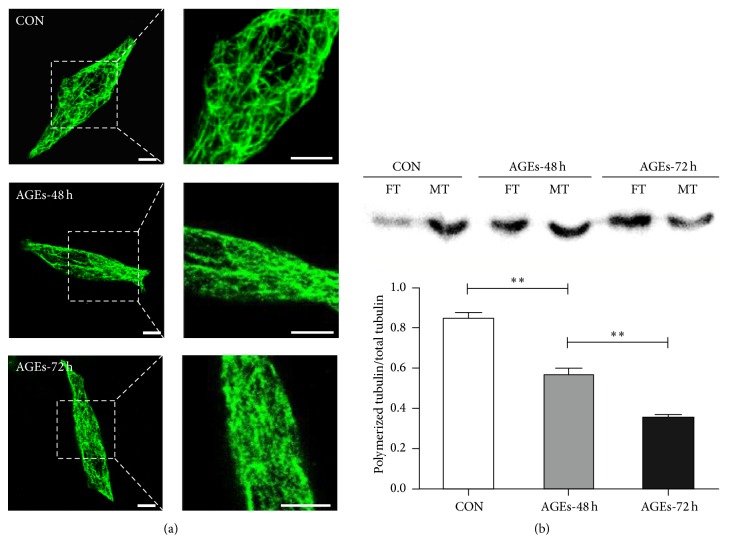
The effect of AGEs on microtubule dynamics in INS-1-3 cells. (a) Immunostaining images of *α*-tubulin (green) in INS-1-3 cells incubated with or without AGEs. CON: INS-1-3 cells without treatment; AGEs-48 h and AGEs-72 h: INS-cells treated with AGEs (200 *μ*g/mL) for 48 or 72 hours, respectively. Scale bar: 25 *μ*m. The boxed areas are shown at higher magnification in the right panels to illustrate details of microtubules. (b) The immunoblots of tubulin from soluble and insoluble fractions of INS-1-3 cells (CON) and AGEs-treated cells. Soluble and insoluble fractions containing free tubulin (FT) and polymerized tubulin (MT) were probed with anti-*α*-tubulin antibody. One of the representative results is shown. Statistical analysis was performed based on three immunoblotting results. The polymerization status of tubulins is presented as the percentage of polymerized tubulin divided by the total tubulin content (sum of densitometric values of free and polymerized tubulins). Data are shown as mean ± SD. ^*∗∗*^ *p* < 0.01.

As exposure to AGEs led to an increase of insulin synthesis but a decrease of glucose-stimulated insulin secretion in INS-1-3 cells, we hypothesized that the decreased insulin secretion is due to defects following insulin synthesis, such as the intracellular transportation of insulin-containing vesicles. As microtubule network plays important role in this process, we tested whether an exposure to AGEs affects the microtubule dynamics. We imaged the microtubule network in INS-1-3 cells by immunostaining and confocal fluorescence microscopy. The microtubules were visualized as a filamentous network in untreated INS-1-3 cells, but the filamentous microtubules were disrupted after AGE treatment. Discontinued tubulin plaques appeared after treatment with AGEs for 48 hours, and even more of those depolymerized tubulin plaques were detected at 72 hours (Figure 3(a)). We postulated this morphological change of microtubule network may be due to changes of its dynamics. To address this question, we separated and quantified the soluble and insoluble fractions of microtubules from INS-1-3 cells treated with or without AGEs. The microtubule filaments are insoluble, whereas the depolymerized tubulins are soluble. Therefore, the ratio of polymerized tubulin to the total tubulin amount equals the ratio of tubulin in the insoluble fraction of INS-1-3 cells to that of the sum of soluble and insoluble fractions. Notably, about 84% of tubulin was polymerized in untreated cells. After 48 hours of AGEs treatment, only 56% of tubulin was found in polymerized form. After 72 hours of AGEs treatment, this number dropped further to 35% (Figure 3(b)). Exposure to AGEs led to a time-dependent decrease of microtubule filaments. Instead, depolymerized tubulin increased in the AGEs-treated INS-1-3 cells.

### 3.4. AGEs Induce Microtubule Depolymerization through p38/MAPK Pathway

**Figure 4:**
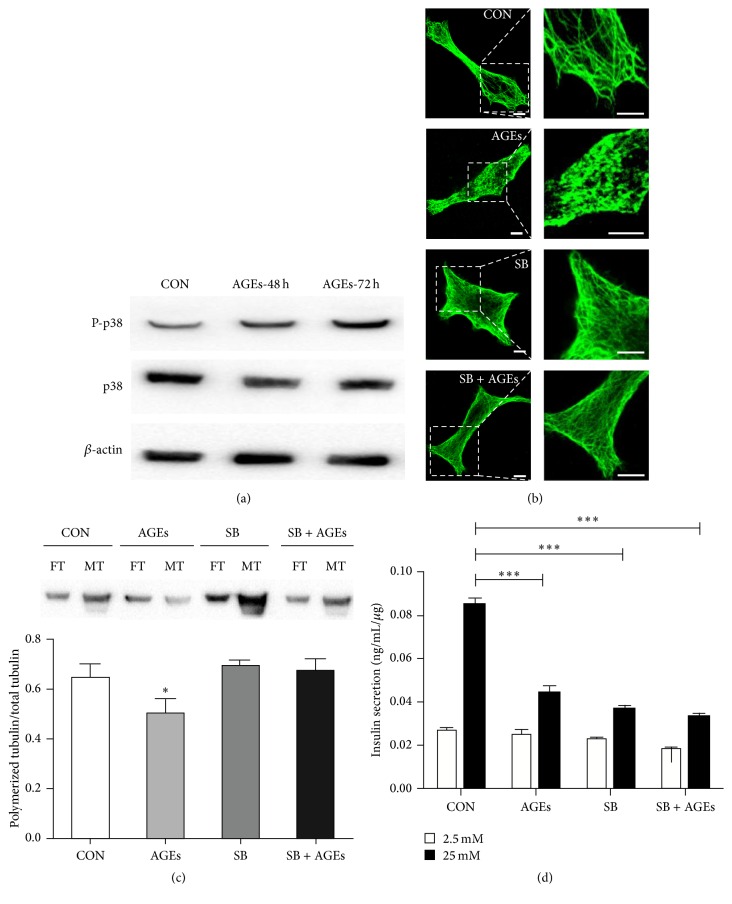
AGEs activate p38/MAPK signaling pathway that disrupts microtubule dynamics. (a) Immunoblots of INS-1-3 cells cultured without (CON) or with 200 *μ*g/mL AGEs for 48 hours (AGEs-48 h) or 72 hours (AGEs-72 h). The levels of phosphorylated and total p38/MAPK were immunoblotted by phosphospecific and general anti-p38 antibodies, respectively. *β*-actin was used as loading control. (b) Immunostaining images of *α*-tubulin (green) in INS-1-3 cells. INS-1-3 cells were cultured without (CON) or with 200 *μ*g/mL AGEs in the absence or presence of 50 *μ*M SB202190 (SB) for 48 hours. The boxed areas are shown at higher magnification in the right panels to illustrate the details of the microtubules. Scale bar: 25 *μ*m. (c) Quantification of free tubulin (FT) and polymerized tubulin (MT) fractions of INS-1-3 cells by immunoblots. One of the representative results is shown. Statistical analysis was performed based on three immunoblotting results. The polymerization status of tubulin is presented as the percentage of polymerized tubulin divided by the total tubulin content. ^*∗*^ *p* < 0.05; AGE group versus CON, SB, or SB + AGEs group. (d) Relative insulin secretion from INS-1-3 cells in response to 2.5 mM or 25 mM glucose, normalized to the total insulin content, measured by ELISA. Data were derived from three independent experiments and shown as mean ± SD. ^*∗∗∗*^ *p* < 0.001 versus control.

As p38/MAPK can be activated by AGEs [8] and phosphorylation of p38/MAPK regulates microtubule polymerization in endothelial cells [20, 21], we tested whether p38/MAPK was activated by AGEs treatment on our cell model. Phosphorylated p38/MAPK (P-p38) levels in AGEs-treated INS-1-3 were quantified by immunoblot. Shown in Figure 4(a), even though the total p38/MAPK amount slightly decreased, its activated form, the P-p38/MAPK, was elevated in both cells treated with AGEs for 48 and 72 hours. To test whether AGEs-induced microtubule depolymerization was due to p38/MAPK activation, we used a pharmacological inhibitor of p38/MAPK, SB202190, to treat INS-1-3 cells exposed to AGEs and analyzed its effect on the microtubule dynamics. Confirmed by both immunostaining images of microtubule network and biochemical quantification of the ratio of polymerized tubulin to total tubulin, treatment with SB202190 promoted microtubule polymerization and recovered the polymerized tubulin level damaged in the AGEs-treated cells (Figures 4(b) and 4(c)). This suggests that AGEs-induced microtubule depolymerization is due to p38/MAPK activation. Further, the glucose-stimulated insulin secretion was analyzed in cells that were treated with SB202190. Interestingly, treatment with SB202190 to cells without AGEs exposure reduced insulin secretion upon glucose stimulation, compared to untreated cells. Treatment with SB202190 to AGEs-exposed cells failed to recover the AGEs-impaired glucose-stimulated insulin secretion (Figure 4(d)). Another p38/MAPK inhibitor SB203580 showed similar results as SB202190. It reduced insulin secretion when used alone and could not rescue AGEs-impaired GSIS (Figure S1 in Supplementary Material available online at http://dx.doi.org/10.1155/2016/9073037). In summary, these data showed that a complete inhibition of p38/MAPK-mediated microtubule depolymerization cannot rescue the AGEs-impaired GSIS.

## 4. Discussion

T2DM is a progressive disease due to insulin resistance and a gradual deterioration of *β*-cell function. Many studies have suggested that *β*-cell dysfunction plays an important role in the development of T2DM, which is associated with glucose metabolic disorder [27]. Inadequate production of insulin leads to chronic hyperglycemia, resulting in accelerated formation and accumulation of AGEs [1]. Emerging studies have demonstrated that AGEs can decrease cell viability, impair the insulin secretion, and induce apoptosis of *β*-cells [4–9]. In this study, we found that AGEs did not affect the insulin synthesis in a *β*-cell model but impaired GSIS by disturbing the microtubule dynamics via activation of p38/MAPK signaling.

The impairment of GSIS by AGEs has been reported in *β*-cell lines [5, 6, 9] and isolated islets [7, 9, 28]. Some studies claimed that AGEs impaired the GSIS through suppression of insulin synthesis [5, 6]. However, we discovered that AGEs increased, instead of decreased, insulin expression in INS-1-3 cells. The reason for this discrepancy was not clear and needed further investigation, but our data inferred that, besides affecting insulin synthesis that may lead to impaired insulin secretion, exposure to AGEs may hamper other posttranslational steps, such as transport of insulin-containing vesicles from ER to the plasma membrane.

Glucose stimulates insulin exocytosis in two phases: the first phase is fusion of a ready-releasable pool of plasma membrane-bound insulin granules; the second phase is prolonged insulin secretion with insulin granules trafficking along microtubules to the cell surface from intracellular storage pools to response to the sustained stimulation [10, 11]. In both the first and second phases of insulin secretion, microtubules play an important role, which act as tracks to guide the insulin-containing vesicles to be delivered to the plasma membrane. The dynamic equilibrium between microtubule polymerization and depolymerization is essential to support insulin-granule mobilization in pancreatic *β*-cells. Disturbance of this balance by microtubule depolymerization or stabilization agents reduce insulin secretion [13, 14]. Depolymerization of microtubules leads to reduced insulin granular movements in *β*-cells supported by cinematographic evidences [29–32]. We observed that microtubules were depolymerized after AGEs treatment in a time-dependent manner, which may explain the impairment of insulin secretion in our INS-1-3 cells, as the intracellular insulin content was not reduced.

In T2DM conditions, p38/MAPK can be activated by high concentrations of glucose and AGEs and may play important roles in the pathogenesis [8, 18, 19]. Previous reports have shown that p38/MAPK regulates microtubule stabilization and the inhibitor of p38/MAPK promotes the microtubule polymerization [20, 21]. Our results demonstrated that the phosphorylated p38/MAPK was significantly increased after treatment with AGEs, and pharmacological inhibition of p38/MAPK signaling recovered the AGEs-disrupted microtubule network. This result suggests the AGEs-activated p38/MAPK leads to depolymerized microtubules. Correlated with the result that AGEs-treated cells showed impaired glucose-stimulated insulin release, we proposed that AGEs activated p38/MAPK and damaged the dynamic equilibrium between polymerization and depolymerization of the microtubule cytoskeleton, tending to keep a persistent depolymerized state, resulting in defective GSIS. Inhibition of p38/MAPK activation could not rescue the AGEs-impaired GSIS. One possible reason was that a complete inhibition of p38/MAPK may overreverse the dynamics of microtubule cytoskeleton, tending to keep a persistent polymerized state; thus, the dynamic equilibrium of microtubule was still disordered and the level of insulin secretion could not be reverted. This could also explain why adding of the inhibitor alone already reduced insulin secretion, just as drugs depolymerizing microtubules or stabilizing microtubules both impair GSIS [10, 13, 14]. Another reason might be that AGEs interfered with other processes in insulin secretion, for example, the actin cytoskeleton-mediated initiation phase of insulin-granule release, making the regulation of microtubule dynamics not sufficient for GSIS rescue, for which further investigations were needed.

## 5. Conclusions

In this study we found that exposure to AGEs impaired insulin secretion in a pancreatic *β*-cell model. This impairment is due to a disturbance of microtubule dynamics via activated p38/MAPK signaling. For T2DM therapy, the cytotoxicity of AGEs cannot be neglected due to its detrimental damage to the pancreatic *β*-cells. To preserve *β*-cells, future therapeutic efforts should be aimed at reducing the serum level of AGEs or fine-tuning the AGEs-induced activation of downstream signaling pathways in diabetes patients.

## Supplementary Material

Fig S1. p38/MAPK inhibitor SB203580 cannot rescue AGEs-impaired GSIS.
