# Supplementary material for: Advanced Glycation End Products Impair Glucose-Stimulated Insulin Secretion of a Pancreatic β-Cell Line INS-1-3 by Disturbance of Microtubule Cytoskeleton via p38/MAPK Activation
Source: J Diabetes Res. 2016 Aug 22;2016:9073037. doi: 10.1155/2016/9073037 (PMC5011238; doi:10.1155/2016/9073037)

**Fig S1. p38/MAPK inhibitor SB203580 cannot rescue AGEs-impaired GSIS.**

INS-1-3 cells were cultured in different conditions for 48 hours before the GSIS assay. CON, without AGEs or SB203580; AGEs, AGEs alone (200 μg/ml); SB2.5, SB203580 alone (2.5 μM); AGEs+SB2.5, AGEs + SB203580 (2.5 μM); SB10, SB203580 alone (10 μM); AGEs+SB10, AGEs + SB203580 (10 μM). Relative insulin secretion from INS-1-3 cells in response to 2.5 mM or 25 mM glucose was calculated as the secreted insulin amount normalized to the total insulin amount, measured by ELISA. Data were derived from three independent experiments and shown as mean±SD. ****p*<0.001 *vs*. control; ***p*<0.01 *vs*. control.

**Fig S1.**


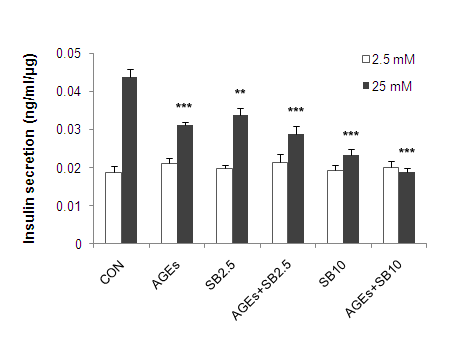

Supplement: Supplementary file 1 — Fig S1. p38/MAPK inhibitor SB203580 cannot rescue AGEs-impaired GSIS. [file 9073037.f1.docx]
